# Supplementary material for: Multilayer Network Analysis of European Regional Flows
Source: Entropy (Basel). 2025 Sep 19;27(9):978. doi: 10.3390/e27090978 (PMC12469737; doi:10.3390/e27090978)
Supplement: Supplementary file 1 [file entropy-27-00978-s001.zip › entropy-3700037-supplementary.pdf]

Supplementary Information of the paper: Multilayer Network Analysis of European Regional Flows

Emanuele Calò, Angelo Facchini

1 Data

Detailed information on the data sources and processing methodologies used for harmonization and estimation of missing values can be found in [2].

1.1 Population

Total population on January 1st, sourced from [1]. Manual additions were made for Mariotte (France) and Ireland for specific years.

We normalize the migration flows by dividing them by the population of the origin region to address a potential bias. According to the gravity model in migration studies, regions with larger populations tend to have higher absolute numbers of incoming and outgoing migrants. By normalizing the flows, we eliminate this population size bias, allowing for a more accurate comparison of regions' relative contributions to migration patterns. This approach is also applied to other types of flows in our study, ensuring consistent analysis across different flow categories.

1.2 GDP

Gross Domestic Product at current market prices (million euros), sourced from [1]. 2011 values were used for regions lacking 2010 data. Manual additions were made for Iceland and Liechtenstein.

2 Methods

We configure the Infomap algorithm with the following parameters:

- two_level=False: Clusters the optimal number of nested modules, accommodating both country-level clusters and single-region clusters.
- num_trials=100: Number of outer-most loops to run before selecting the best solution.
- flow_model='rawdir': Determines node visitation rates based on the given direction and weight of edges, without using a PageRank algorithm.

– entropy_corrected=True: Corrects for negative entropy bias in small samples (many modules).
– multilayer_relax_rate=0.15: Probability of relaxing the constraint to move only within the current layer (default value).

We applied the Infomap algorithm to the multiplex network structure to identify communities. In this analysis, it is possible for a region to be assigned to multiple communities across different layers. To resolve such cases and provide a definitive community assignment, we employed a frequency-based approach. Specifically, each region was ultimately assigned to the community in which it appeared most frequently across all layers.

3 Results

3.1 Network properties

Table S1 and Table S2 present the network statistics across European NUTS-2 regions from 2010 to 2018. For each flow type, we report the number of nodes, which indicates the participating NUTS-2 regions in the network, as well as the number of edges, representing the connections between these regions where a connection signifies a non-zero flow. Additionally, we calculate the density of each network as the ratio of actual connections to the total possible connections, providing insight into how interconnected the regions are within each flow type.

Figure S1 and S2 depict the relationship between in-strength and out-strength for all flow types in 2010 and 2018, respectively. These scatter plots reveal notable differences in correlations between various flow types, which remain consistent across both years. For instance, in 2010, the Spearman correlation coefficients range widely from 0.16 to 0.94, indicating diverse patterns of association be- tween inflows and outflows across different domains. This substantial variation in correlations persists in 2018, suggesting that the underlying structures of these regional flow networks maintain their distinct characteristics over time.

Figure S3 presents the WANNS^{in,out} for the empirical networks alongside 50 realizations drawn from the null model ensembles for FDI, Remittances, and Passengers in 2010.

Figure S4 focuses specifically on the Migration flow type in 2010, showing the relationship between the WANNS^{in,out} and the in-strength for ensemble copies. This visualization helps to understand the assortativity patterns in the migration network, revealing how regions with higher in-strength tend to receive flows from regions with higher out-strength.

Figure S5 presents the CCDF for all flow types in 2018. This plot closely re- sembles the CCDF for 2010 shown in the main paper, indicating a remarkable stability in the strength distributions over time. The consistent tail behavior across both years suggests that the potential heavy-tailed relationships and dis- tribution characteristics for different flow types remain largely unchanged. This similarity underscores the persistent nature of the network structure and flow patterns in the European regional system, with minimal alterations in the rela- tive strengths of connections across various domains between 2010 and 2018.

3.2 Pagerank

Figure S6 illustrates the spatial distribution of PageRank centrality values across European regions for Freight, Erasmus, and Passengers in 2010.

Tables 3 through 9 present the top 10 regions ranked by PageRank for various flow types in 2010. For migration flows (Table S3), Oberbayern emerges as the most central region, followed closely by Ile-de-France and Inner London - East. The list is dominated by German regions, highlighting Germany's significance in European migration patterns. Tourism flows (Table S4) show Cataluña as the top-ranked region, with Ile-de-France and Jadranska Hrvatska following closely. This ranking reflects the popularity of Mediterranean coastal regions for tourism. In freight transport (Table S5), Lombardia leads, followed by Zuid-Holland and Emilia-Romagna, underscoring the importance of industrial and port regions in goods movement. For Erasmus student exchanges (Table S6), Ile-de-France ranks first, followed by Comunidad de Madrid and Andalucía, indicating the attractiveness of these regions for international students. In Foreign Direct In- vestment (FDI) flows (Table S7), Noord-Holland tops the list, with Ile-de-France and Comunidad de Madrid following, reflecting the financial importance of these regions. Passenger flows (Table S8) are dominated by Ile-de-France, with a sig- nificant lead over Comunidad de Madrid and Inner London - West, highlighting Paris's role as a major transportation hub. Finally, for remittance flows (Table S9), Ile-de-France again leads, followed by Cataluña and Luxembourg, indicat- ing the economic significance of these regions for international money transfers. These rankings collectively demonstrate the varied roles that different regions play in different types of flows, with some regions, particularly Ile-de-France, showing high centrality across multiple networks.

Figure S7 demonstrates the relationship between PageRank and in-strength across all flow types in 2010. The analysis reveals strong correlations for most flow types, with both Pearson and Spearman correlation coefficients exceeding 0.74. This indicates a robust association between a region's centrality and the volume of incoming flows for most networks. However, the Freight network stands out as an exception, exhibiting a notably weaker correlation.

Figure S8 illustrates the top 8 and bottom 8 slopes of PageRank trends for Mi- gration, highlighting regions with notably increasing or decreasing centrality. Interestingly, London stands out among the top increasing trends, despite expe- riencing a noticeable dip in 2016, likely attributable to the Brexit referendum. This overall upward trajectory, even in the face of such a significant political event, underscores London's resilience and enduring importance as a migration hub.

Figure S9 displays the correlations between single-layer PageRank values and the multiplex PageRank for various flow types in 2010. The Spearman correlation coefficients range from 0.54 to 0.73, indicating moderate positive relationships between individual layer centralities and the overall multiplex centrality.

3.3 Community Detection

Figure S10 illustrates the relationship between the number of communities and the multilayer relax rate for 2010, showing that the range around the standard value (from 0.1 to 0.2) yields a relatively stable number of communities, varying from 70 to 89. Generally, increasing the relax rate r leads to a higher number of communities, until it reaches an extremely high value (0.9), at which point the number of communities sharply decreases to around 10.

Table S10 presents the results of the Infomap community detection. analysis.

References

1. Espon database portal. https://database.espon.eu/, accessed: 2024-06-17
2. Irie. https://gis-portal.espon.eu/arcgis/apps/sites/#/irie-hub?, accessed: 2024-10-29

Layer	Year	Nodes	Edges	Density (%)
Migration	2010	297	70797	80.53
Migration	2011	297	71535	81.37
Migration	2012	297	72459	82.42
Migration	2013	297	74502	84.75
Migration	2014	297	75342	85.70
Migration	2015	297	75580	85.97
Migration	2016	297	75955	86.40
Migration	2017	297	76044	86.50
Migration	2018	297	76281	86.77
Tourism	2010	297	87635	99.68
Tourism	2011	297	87649	99.70
Tourism	2012	297	87652	99.70
Tourism	2013	297	87665	99.72
Tourism	2014	297	87662	99.72
Tourism	2015	297	87669	99.72
Tourism	2016	297	87686	99.74
Tourism	2017	297	87682	99.74
Tourism	2018	297	87692	99.75
Freight	2010	297	46695	53.12
Freight	2011	297	47315	53.82
Freight	2012	297	46820	53.26
Freight	2013	297	46663	53.08
Freight	2014	297	46703	53.12
Freight	2015	297	44742	50.89
Freight	2016	297	43628	49.63
Freight	2017	297	43326	49.28
Freight	2018	297	42587	48.44
Erasmus	2010	265	21551	30.80
Erasmus	2011	264	22094	31.82
Erasmus	2012	270	23405	32.22
Erasmus	2013	272	24027	32.60
Erasmus	2014	274	24576	32.85

Table S1: Network statistics for Migration, Tourism, Freight, and Erasmus.

Layer	Year	Nodes	Edges	Density (%)
FDI	2010	292	24872	29.27
FDI	2011	292	25028	29.45
FDI	2012	292	25178	29.63
FDI	2013	292	25309	29.79
FDI	2014	292	25360	29.85
FDI	2015	292	25422	29.92
FDI	2016	292	25483	29.99
FDI	2017	292	25521	30.03
FDI	2018	292	25441	29.94
Passengers	2010	297	12144	13.81
Passengers	2011	297	12486	14.20
Passengers	2012	297	12547	14.27
Passengers	2013	297	12604	14.34
Passengers	2014	297	12649	14.39
Passengers	2015	297	12588	14.32
Passengers	2016	297	12716	14.46
Passengers	2017	297	12849	14.62
Passengers	2018	297	12998	14.79
Remittances	2010	297	81375	92.56
Remittances	2011	297	81355	92.54
Remittances	2012	297	81410	92.60
Remittances	2013	297	81243	92.41
Remittances	2014	297	81172	92.33
Remittances	2015	297	81016	92.16
Remittances	2016	297	81236	92.41
Remittances	2017	297	81250	92.42
Remittances	2018	297	81250	92.42
Horizon2020	2015	287	7482	9.12
Horizon2020	2016	284	7135	8.88
Horizon2020	2017	288	6899	8.35
Horizon2020	2018	279	6475	8.35

Table S2: Network statistics for FDI, Passengers, Remittances, and Horizon2020.

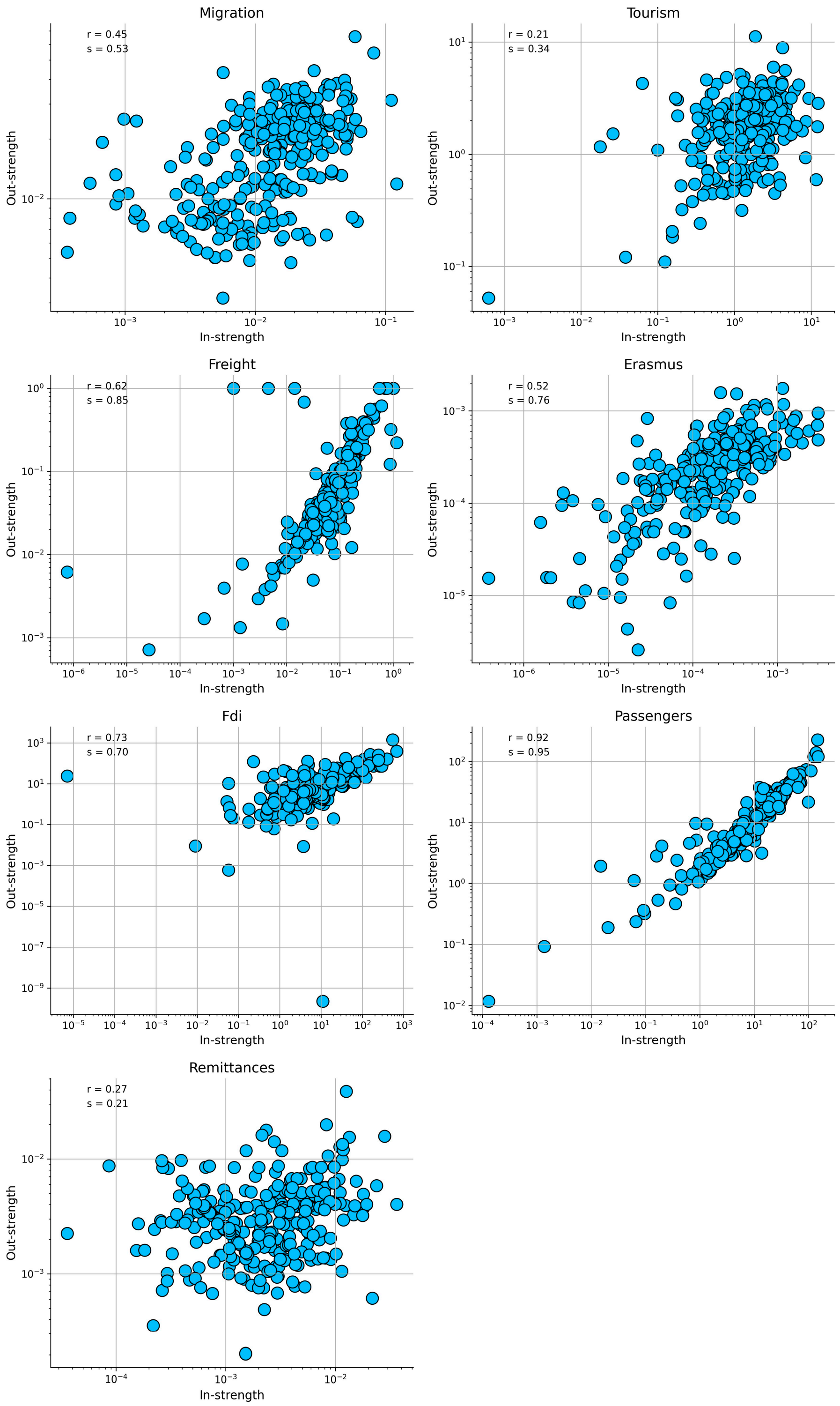

Figure S1: In-strength VS out-strength for the year 2010.

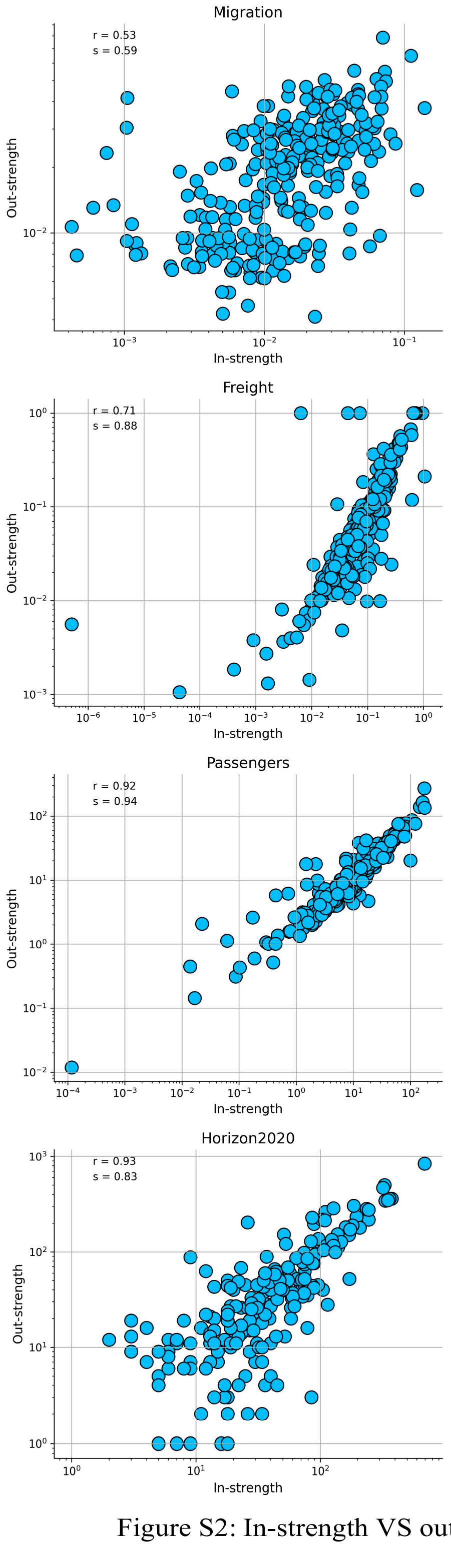

Figure S2: In-strength VS out-strength for the year 2018.

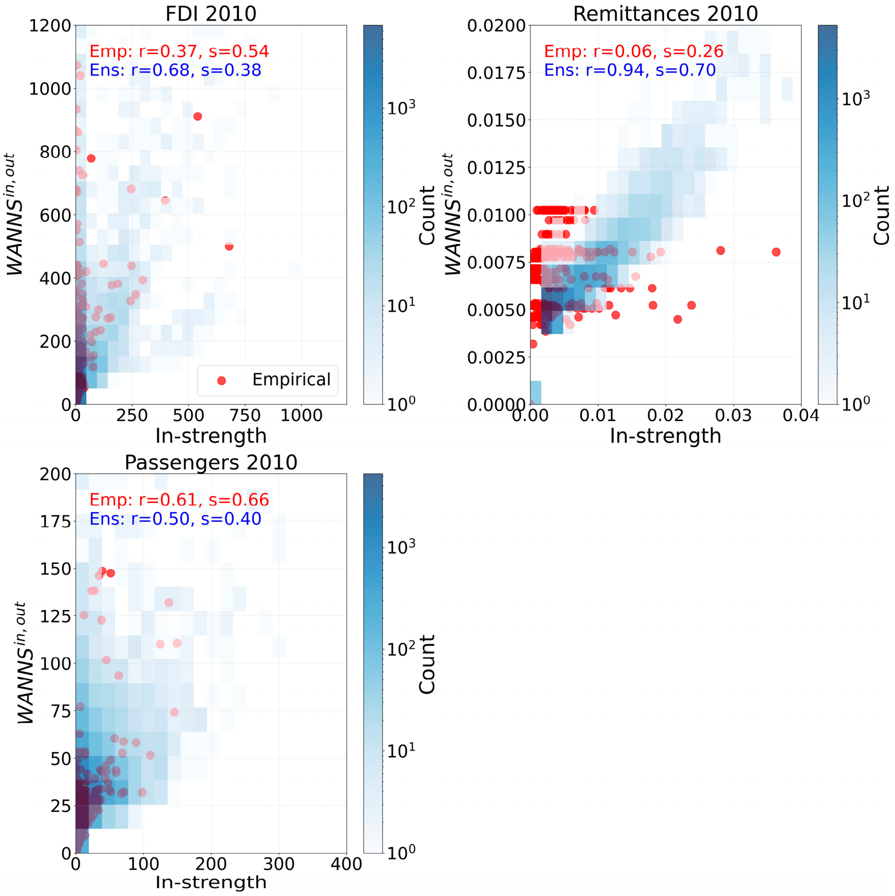

Figure S3: Comparison of empirical and ensemble WANNS for FDI, Remittances and Passengers in 2010. Each subplot shows the relationship between in-strength and WANNS^{in,out} values. The red points indicate empirical data, while the blue histogram represents the distribution of ensemble results. Correlation coefficients (Pearson's r and Spearman's s) are displayed for both empirical and ensemble data.

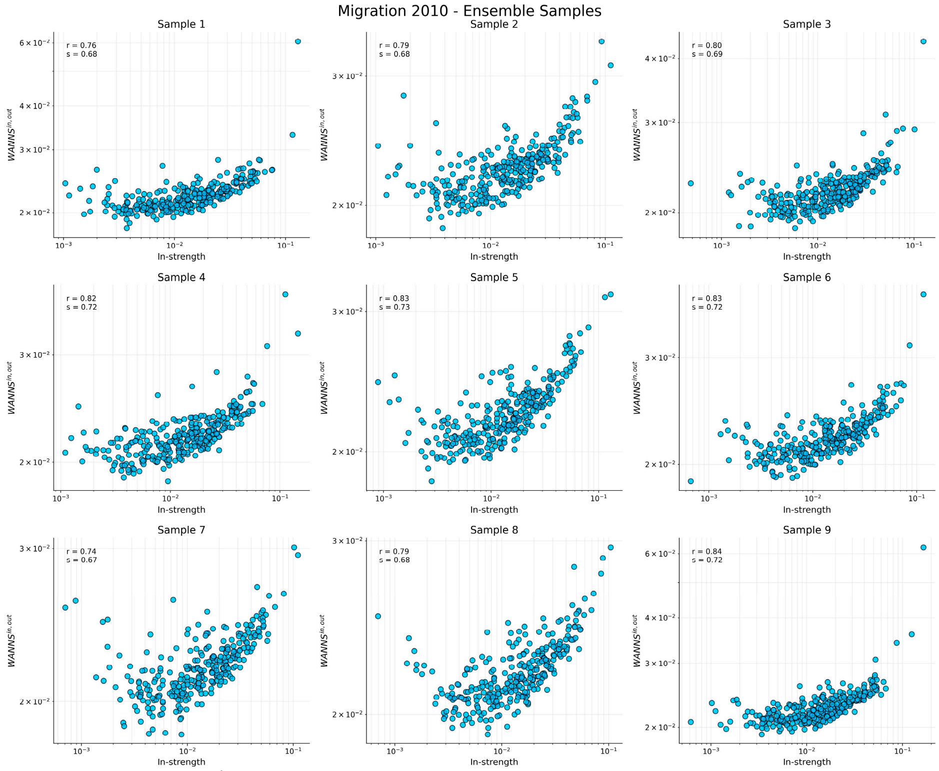

Figure S4: WANNS^{in,out} VS in-strength for ensemble copies for Migration the year 2010.

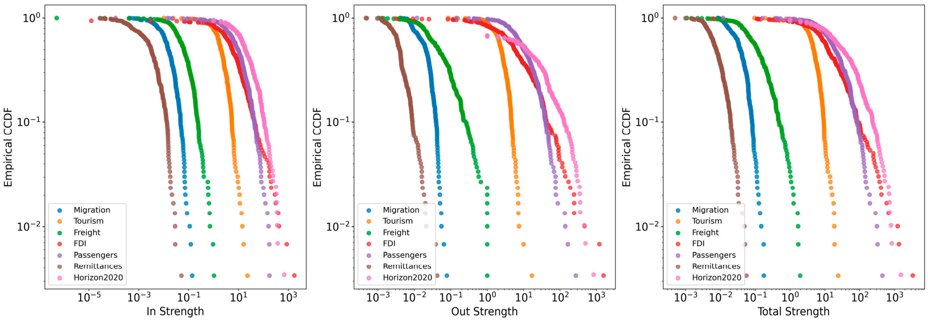

Figure S5: Complementary cumulative distribution function for the year 2018.

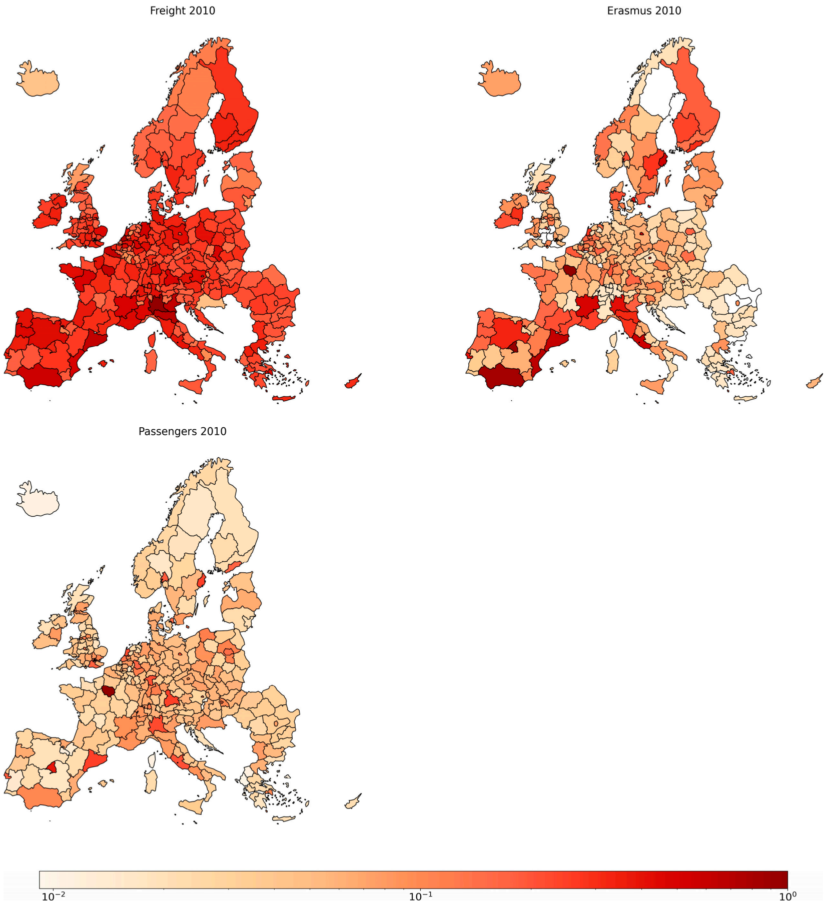

Figure S6: PageRank for Freight, Erasmus, and Passengers in 2010. Colors are dis- played on a logarithmic scale, with values normalized such that the region with the highest centrality is set to 1.

Region	PageRank
Oberbayern	1.0000
Ile-de-France	0.8087
Inner London - East	0.6598
Darmstadt	0.6441
Stuttgart	0.6259
Düsseldorf	0.6252
Berlin	0.6167
Köln	0.6105
Attiki	0.6045
Lombardia	0.6024

Table S3: Top 10 regions by PageRank for Migration in 2010.

Region	PageRank
Cataluña	1.0000
Ile-de-France	0.9793
Jadranska Hrvatska	0.9237
Andalucía	0.7983
Rhône-Alpes	0.6389
Provence-Alpes-Côte d'Azur	0.6367
Veneto	0.5567
Comunidad Valenciana	0.5176
Lombardia	0.4910
Comunidad de Madrid	0.4865

Table S4: Top 10 regions by PageRank for Tourism in 2010.

Region	PageRank
Lombardia	1.0000
Zuid-Holland	0.7975
Emilia-Romagna	0.6890
Veneto	0.6428
Cataluña	0.6336
Ile-de-France	0.6021
Nord-Pas de Calais	0.5995
Andalucía	0.5737
Weser-Ems	0.5091
Comunidad Valenciana	0.5006

Table S5: Top 10 regions by PageRank for Freight in 2010.

Region	PageRank
Ile-de-France	1.0000
Comunidad de Madrid	0.9202
Andalucía	0.8484
Comunidad Valenciana	0.6656
Cataluña	0.6186
Berlin	0.5657
Lazio	0.5479
Stockholm	0.5269
Rhône-Alpes	0.4794
Hovedstaden	0.4272

Table S6: Top 10 regions by PageRank for Erasmus in 2010.

Region	PageRank
Noord-Holland	1.0000
Ile-de-France	0.6988
Comunidad de Madrid	0.5451
Lombardia	0.4559
Bucureşti - Ilfov	0.4537
Luxembourg	0.4333
Eastern and Midland	0.4220
Région de Bruxelles-Capitale	0.3791
Zuid-Holland	0.3750
Warszawski stołeczny	0.2924

Table S7: Top 10 regions by PageRank for FDI in 2010.

Region	PageRank
Ile-de-France	1.0000
Comunidad de Madrid	0.3822
Inner London - West	0.3775
Inner London - East	0.3187
Stockholm	0.2466
Oberbayern	0.2449
Cataluña	0.2446
Berlin	0.2232
Noord-Holland	0.2197
Lombardia	0.2186

Table S8: Top 10 regions by PageRank for Passengers in 2010.

Region	PageRank
Ile-de-France	1.0000
Cataluña	0.9165
Luxembourg	0.7257
Comunidad de Madrid	0.7015
Vidurio ir vakarų Lietuvos regionas	0.6988
Prov. Antwerpen	0.5824
Rhône-Alpes	0.5389
Latvija	0.4810
Prov. Oost-Vlaanderen	0.4793
Comunidad Valenciana	0.4563

Table S9: Top 10 regions by PageRank for Remittances in 2010.

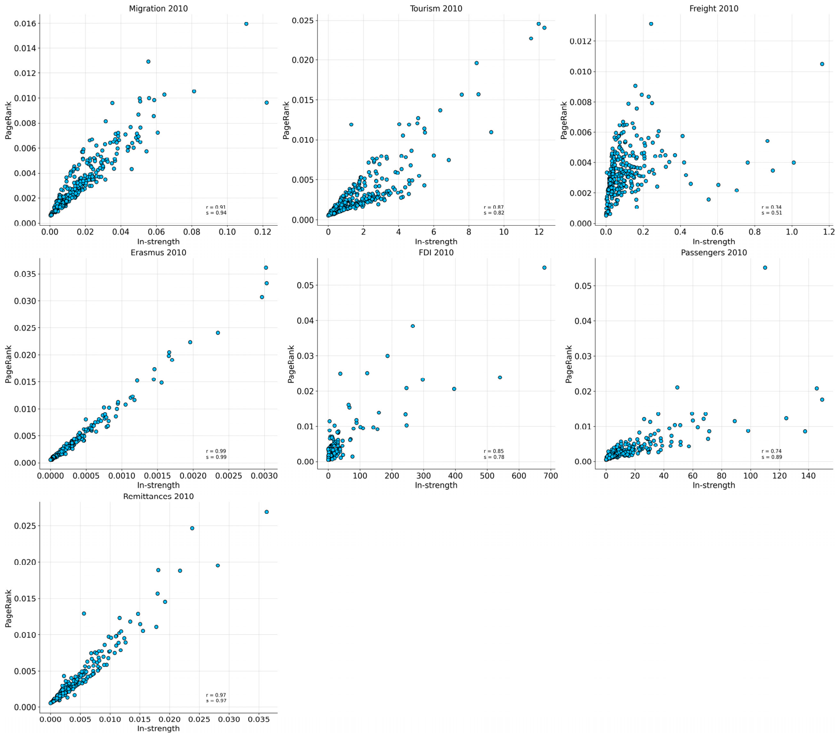

Figure S7: PageRank VS in-strength for the year 2010.

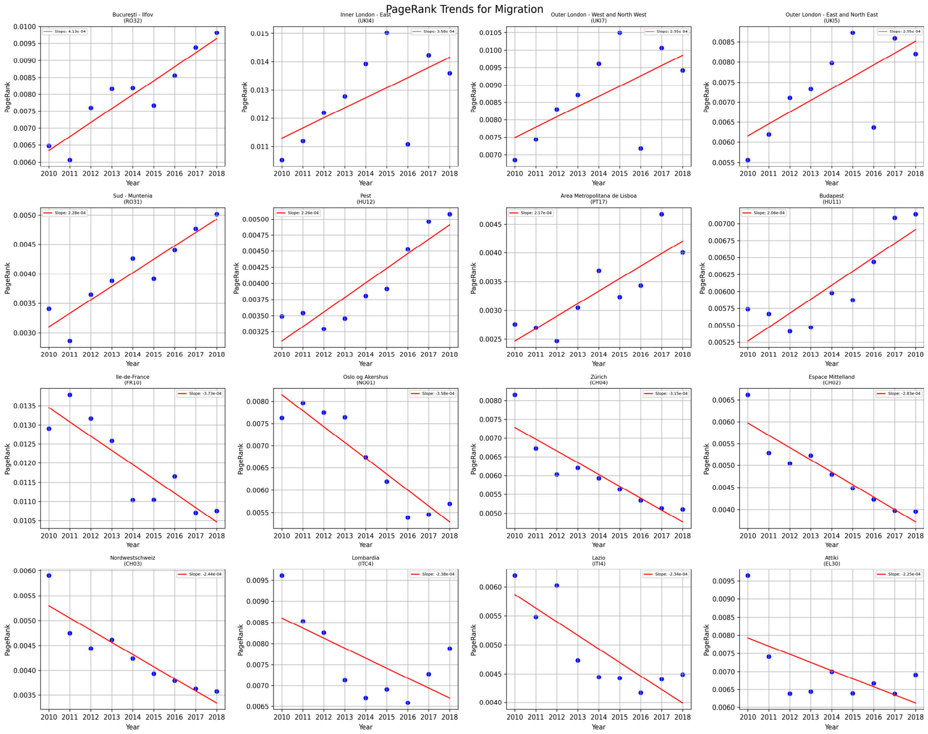

Figure S8: Top 8 and bottom 8 slopes of PageRank Trends for Migration.

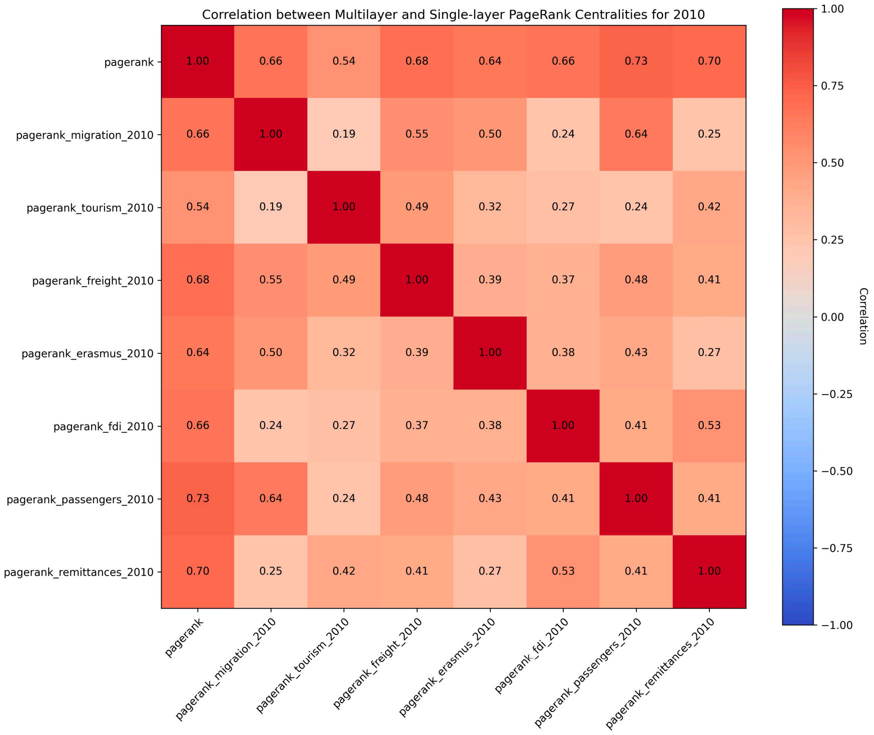

Figure S9: Correlations among single-layer PageRank and multiplex PageRank for 2010.

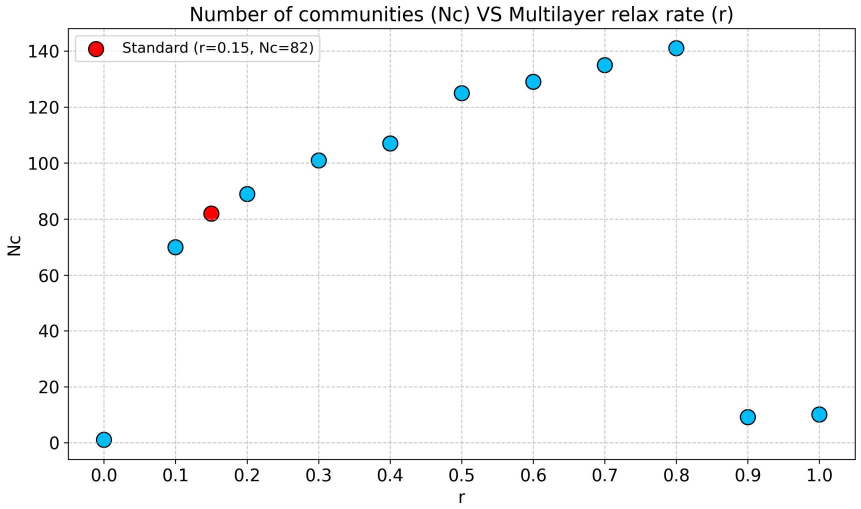

Figure S10: Number of communities VS multilayer relax rate for 2010.

Table S10: Infomap community detection results.

NUTS ID	Community	NUTS ID	Community	NUTS ID	Community	NUTS ID	Community	NUTS ID	Community	NUTS ID	Community
FRM0	1	BE23	4	CY00	8	HU23	14	DE11	23	DE93	40
FR10	1	BE22	4	UKK1	8	HU31	14	DE12	23	UKK4	41
FRL0	1	BE21	4	UKJ4	8	HU33	14	DE14	23	UKK3	41
FRE2	1	BE10	4	UKJ2	8	HU22	14	DE13	23	DE24	42
FRY3	1	NL34	4	UKJ3	8	HU32	14	UKN0	24	DE25	42
FRB0	1	BE25	4	RO11	9	HU12	14	IE04	24	DE94	43
FRK2	1	LU00	4	RO41	9	HU11	14	IE05	24	DED2	44
FRJ1	1	ITC2	5	RO21	9	HU21	14	IE06	24	DEG0	45
FRI3	1	ITC1	5	RO32	9	DK04	15	UKE2	25	FRF1	46
FRI2	1	ITH3	5	RO31	9	DK02	15	UKE3	25	FRY1	47
FRI1	1	ITC4	5	RO42	9	DK03	15	UKE4	25	FRY2	47
FRH0	1	ITI4	5	RO12	9	DK01	15	UKC2	25	DED4	48
FRJ2	1	ITF5	5	RO22	9	DK05	15	UKC1	25	FRF3	49
FRG0	1	ITF6	5	AT11	10	HR03	16	UKE1	25	ES62	50
ES70	2	ITF2	5	AT34	10	MT00	16	DE60	26	FRD2	51
ES64	2	ITI1	5	AT33	10	SI03	16	DE80	26	FRF2	52
ES63	2	ITH5	5	AT12	10	SI04	16	DEF0	26	ITF3	53
ES61	2	NL32	6	AT13	10	HR04	16	DE27	27	DE73	54
ES53	2	NL11	6	AT21	10	NO06	17	DE21	27	DEA4	55
ES51	2	NL12	6	AT22	10	NO02	17	UKD1	28	ITC3	56
ES42	2	NL13	6	AT32	10	NO03	17	UKD6	28	FRC1	57
ES23	2	NL21	6	AT31	10	NO05	17	UKD3	28	DEC0	58
ES24	2	NL33	6	SE23	11	NO07	17	UKD4	28	FRY4	59
ES30	2	NL42	6	SE33	11	NO04	17	UKD7	28	FRY5	59
ES41	2	NL31	6	SE21	11	NO01	17	UKM9	29	ITF4	60
ES11	2	NL23	6	SE22	11	EE00	18	UKM5	29	DE23	61
ES52	2	NL22	6	SE12	11	LV00	18	UKM6	29	DE72	62
PL62	3	NL41	6	SE11	11	LT02	18	UKM7	29	DE26	63
PL51	3	CZ06	7	SE32	11	LT01	18	UKM8	29	FRC2	64
PL52	3	SK04	7	FI20	11	CH04	19	DE30	30	DE22	65
PL61	3	CZ02	7	SE31	11	LI00	19	DE40	30	ITG1	66
PL63	3	CZ04	7	EL65	12	CH03	19	UKG3	31	ITI3	67
PL71	3	CZ03	7	EL53	12	CH05	19	UKG1	31	ITH2	68
PL72	3	CZ01	7	EL52	12	CH01	19	UKG2	31	ES22	69
PL81	3	CZ07	7	EL51	12	CH02	19	UKF1	32	ITI2	70
PL92	3	SK01	7	EL54	12	CH06	19	UKF2	32	DEB2	71
PL91	3	SK02	7	EL42	12	BG32	20	UKF3	32	ITH1	72
PL82	3	SK03	7	EL41	12	BG31	20	DE91	33	ITH4	73
PL43	3	CZ05	7	EL30	12	BG42	20	DE92	33	ITG2	74
PL41	3	CZ08	7	EL61	12	BG33	20	DE71	34	FRK1	75
PL42	3	UKH3	8	EL62	12	BG34	20	DEB1	35	PT15	76
PL84	3	UKJ1	8	EL63	12	BG41	20	DEB3	35	FRD1	77
PL21	3	UKK2	8	EL64	12	DEA2	21	DED5	36	ITF1	78
PL22	3	UKH1	8	EL43	12	DEA3	21	DEE0	36	ES43	79
BE35	4	UKH2	8	PT30	13	DEA5	21	ES12	37	ES13	80
BE34	4	UKI3	8	PT11	13	DEA1	21	ES21	37	CH07	81
BE33	4	UKI4	8	PT16	13	FI1D	22	UKL2	38	IS00	82
BE32	4	UKI5	8	PT17	13	FI1C	22	UKL1	38		
BE31	4	UKI6	8	PT18	13	FI1B	22	FRE1	39		
BE24	4	UKI7	8	PT20	13	FI19	22	DE50	40		
